# Generation and Application of Fluorescent Anti-Human β2-Microglobulin VHHs via Amino Modification

**DOI:** 10.3390/molecules24142600

**Published:** 2019-07-17

**Authors:** Chundong Huang, Da Li, Jun Ren, Fangling Ji, Lingyun Jia

**Affiliations:** Liaoning Key Laboratory of Molecular Recognition and Imaging, School of Bioengineering, Dalian University of Technology, No.2 Linggong Road, Dalian 116023, Liaoning, China

**Keywords:** VHHs, single domain antibodies, amino modification, protein structures, fluorescent

## Abstract

The functionalization of VHHs enables their application in almost every aspect of biomedical inquiry. Amino modification remains a common strategy for protein functionalization, though is considered to be inferior to site-specific methods and cause protein property changes. In this paper, four anti-β2M VHHs were selected and modified on the amino group by NHS-Fluo. The impacts of amino modification on these VHHs were drastically different, and among all th examples, the modified NB-1 maintained the original stability, bioactivity and homogeneity of unmodified NB-1. Specific recognition of VHHs targeting β2M detected by fluorescence imaging explored the possible applications of VHHs. Via this study, we successfully functionalized the anti-β2M VHHs through amino modification and the results are able to instruct the simple and fast functionalization of VHHs in biomedical researches.

## 1. Introduction

Heavy-chain-only antibodies (HcAbs) are a unique type of antibody devoid of light chains, originally found in the serum of camelids [1]. The antigen-binding fragment of HcAbs is solely composed of a single variable domain, referred to as variable domain of the heavy chain of HcAbs (VHHs), single-domain antibody or nanobody. VHHs, only 13~15 kDa in size, are the smallest fully functional antibody fragments, which enables their wide usage in scientific research [2,3,4], medical diagnosis and therapy [5,6,7]. While early applications relied exclusively on the function of the nanobody itself, more recent developments combine the target specificity of nanobodies with the effectiveness of other tags via modifications [8,9], such as fluorescent molecules or radioactive isotopes for applied in immunological detection.

Amino modification is a well-understood and popular method for protein modification. In most VHHs, there are several lysine residues with λ amino groups on the side chain. Therefore, amino modifications of VHHs could achieve high reaction efficiency and improve the effective load of the labels, which is meaningful for improve the antibody-drug conjugate (ADC) efficiency or detection sensitivity. During this process, no extra genetic engineering or enzymes are required [10]. Besides, after decades of developments, there are numerous commercial reagents and ligands available for amino modification.

Many previous studies prepared probes based on VHHs modified on amino groups, and used them for PET/CT imaging, fluorescence microscopy and immunofluorescent staining assays [11,12,13,14]. However, some other studies indicated that random amino modifications may change the structure of VHHs, resulting in a significant reduction or complete loss of antigen binding activity and stability [15,16,17]. Besides, the several isomers contained in the products were difficult to purify and characterize, which discouraged their application [18,19].

The individual difference between VHHs may be the main reason leading to almost completely opposite conclusions about amino modification. However, no research found has focused on how amino modification affects VHHs’ characteristics. As a class of homologous antigen binding protein, huge potential sequence diversity exists in VHH sequences, and studies have fully reflected the diversity of VHHs, including structure, stability, surface charge and isoelectric point [20,21,22,23], resulting in their differences in tolerance to amino modification.

β2-Microglobulin (β2M) is the light chain of the major histocompatibility complex on the cell surface. The accumulation of β2M present in tissue fluid and serum is significantly associated with kidney diseases, bone diseases and various cancers. More importantly, it’s believed to directly lead to dialysis-related amyloidosis (DRA) [24,25,26]. Therefore, the in vitro detection and in vivo imaging of β2M and β2M fibril is significantly important.

For that, four anti-β2M VHHs (NB-1, NB-2, NB-3 and NB-4) were selected and modified with defined stoichiometric ratios of NHS-Fluo on the amino groups, then analyzed and characterized by native polyacrylamide gel electrophoresis (Native-PAGE), intact protein mass spectrometry, circular dichroism and SPR. We found that amino modification affected VHHs to quite varying degrees.

Especially, the affinity of NB-1 was only slightly reduced from 6 × 10^−8^ M to 3 × 10^−7^ M and the denaturation temperature was almost unreduced. The mass spectrometry result showed that the number and location of fluorescein NB-1 modification were relatively narrow when compared with NB-2, NB-3 and NB-4. Further fluorescence imaging in laser scanning confocal microscopy proved the modified NB-1 can be used in microscopy or disease diagnosis. Obviously, amino modification might be a simple and efficient method for some VHHs. Meanwhile, further research is necessary to find the underlying rules. The aim of this paper was to describe how amino modification affects the model system of selected VHHs, and gives a counterexample where amino group modified VHHs retains as good characteristics as unmodified ones.

## 2. Results and Discussion

### 2.1. Amino Group Distribution in Four VHHs

**Figure 1:**
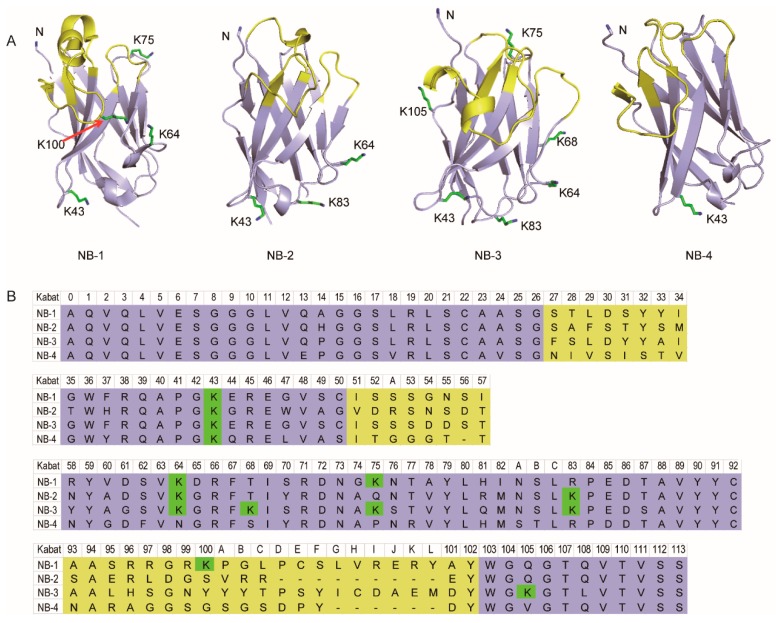
Stereoscopic structure, amino group distribution schematic (**A**) and sequences (**B**) of the selected four VHHs. The amino acid was numbered in Kabat scheme (n) instead of the real sequence number. “K” present lysine residue, “Kn” present the lysine residues located in Kabat scheme Hn, and “N” presents the N-terminal amino group.

Homology-modeling visualized the structure character and lysine residues distribution in three-dimensional space (Table 1 and Figure 1). The result showed the sites of lysine residues (in Kabat scheme) were relatively conservative, but highly significant differences in the amino group number were observed. Briefly, NB-4 has only one lysine residue, but NB-3 has six lysine residues. Most of those lysine residues are located in the loops, not involved with the antigen binding region. Especially, there was a lysine residue in the CDR3 of NB-1 (K100), which is usually considered will strictly damage the affinity of VHH if it’s modified. It can thus be concluded to the most important premise of this study—that is, huge difference still exists between each VHH.

### 2.2. Isomers Composition of the Modified VHHs

**Figure 2:**
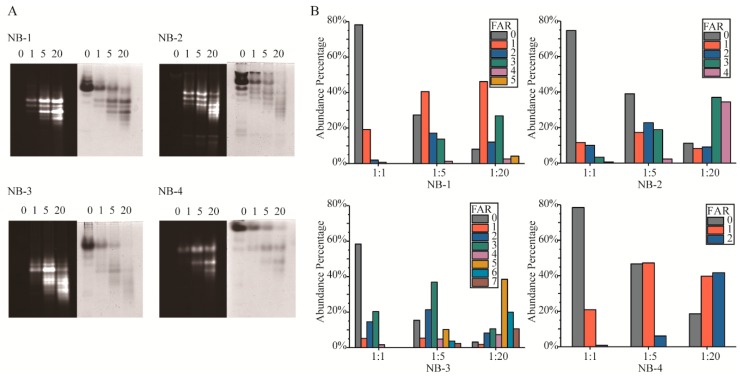
Analysis of isomers in the modified VHHs. (**A**) Native polyacrylamide gel electrophoresis (Native-PAGE) analysis of the modified VHHs. The gels were first visualized under ultraviolet light to get fluorescence signal (gels with black background), after that the gels were stained with Coomassie brilliant blue and visualized white light (gels with white background). Molar ratio (NHS-Fluo:VHH) of each group was labeled upon the lanes. (**B**) Different FAR of isomers in the modified VHH analyzed by LC-MS intact protein analysis. The percentage of isomers was visualized in a percentage accumulation diagram. Molar ratio of each group was labeled upon the histogram.

Amino modification has been criticized for its inevitable generation of heterogeneous products. The fundamental cause of isomers was the multiple amino groups in protein. Native-PAGE (Figure 2A) and LC-MS intact protein analysis (Figure 2B) were performed to detect the Fluo-antibody ratio (FAR) isomers in the modified products. In Native-PAGE, the positive charge on the surface of VHHs decreased with more amino groups modified by NHS-Fluo, resulting in higher fluorescein load isomers that move faster in gels. Unlike the clearly separated number isomers, the position isomers were more likely to form two adjacent bands or a blurry band. In order to describe in depth the FAR isomers, we analyzed the products by LC-MS to detect the percentage of different FAR components (Figure 2B) and calculated the average FAR (Table 2) of the products by intact protein analysis. As expect, it is observed that NB-3 generated most isomers than NB-1, NB-2 and NB-4. Correspondingly, the products of the modified NB-4 were relatively homogeneous. This result was consistent with the amount of amino groups in each VHH.

The results showed that when four selected VHHs were modified with increased molar ratios of NHS-Fluo, higher FAR products were generated, and the average FAR increased gradually. Meanwhile, more kinds of isomers were presented in the mixtures. It could be found that the mean and standard deviation (SD) of FAR of modified products had a positive correlation with the lysine amounts of the VHHs, but the modified products of NB-1 had smaller SD and less FAR that of NB-2 when modified by the same molar ratio of NHS-Fluo, although there were five amino groups in NB-1 and only four in NB-2. This result indicated the modified products of NB-1 were more homogeneous than those of NB-2. Meanwhile, the percentages of different FAR isomers in modified NB-1 were discrete, rather than normally distributed. Specifically in the products modified by 20 fold NHS-Fluo, there were 8.1% unmodified, 46.2% monomodified, 12.1% dimodified, 26.8% trimodified, 2.6% tetramodified and 4.2% pentmodified. This phenomenon was fundamentally caused by the different reactivity of amino groups, the chemical environment and nearby amino acids. Above all, a higher titer of labels could be achieved by amino modification for all of the selected VHHs, which is meaningful for improve the ADC efficiency or detection sensitivity.

### 2.3. Recovery of the Modified VHHs

**Figure 3:**
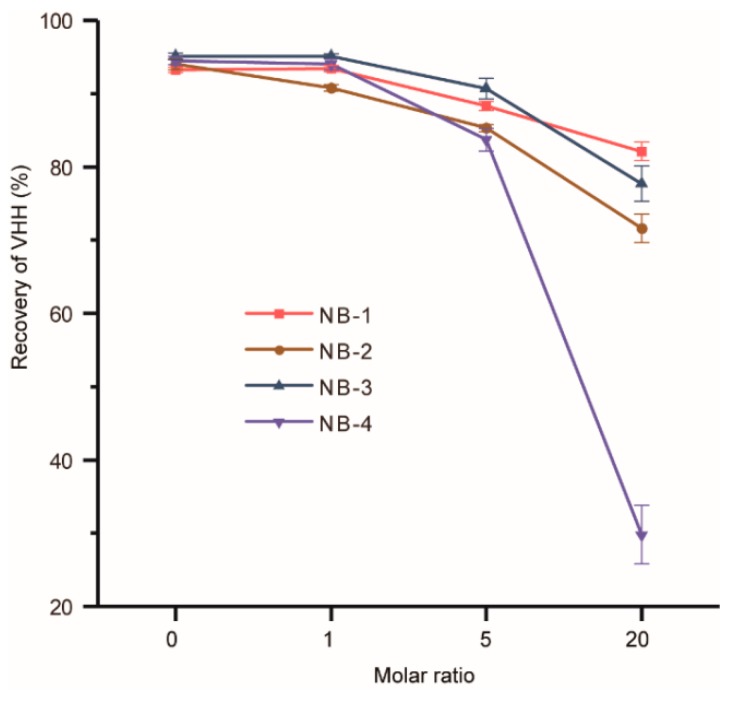
Recovery of the modified VHHs. Four different molar ratios of NHS-Fluo were added individually for modification. Each group had three repetitions, and the average was taking.

Modifications may change the surface charge and the structure of VHHs, resulted in protein aggregation. The recovery of modified VHHs was used to evaluate the stability of four selected VHHs (Figure 3). The groups of the molar ratio at zero were set as negative controls and other three different molar ratios of the recovery of four VHHs decreased to varied degrees. It could be found that the recovery of NB-4 decreased dramatically when modified by higher molar ratios of NHS-Fluo. Surprisingly, the recovery of NB-4 abnormally dropped from 82% (modified by a 5-fold amount of NHS-Fluo) to only 30% (modified by a 20-fold amount of NHS-Fluo), but the recoveries of modified NB-1, NB-2 and NB-3 remained about 80%. Our analysis could not explain precisely why the stability of NB-4 was extremely sensitive to amino modification, but we can make reasonable conjectures that the aggregation was caused by modification, suggesting structural instability compared with NB-1, NB-2 and NB-3. When it is considered that NB-4 has only one lysine residue besides the N-terminal amino group (Figure 1), and K44 also exists in other three VHHs, this phenomenon was more noteworthy.

### 2.4. Affinity of the Modified VHHs

Bioactivities of VHHs were measured to determine whether the modified VHHs retain their function and binding kinetic properties. The samples named as VHH-origin represented VHHs without modification or other treatment, and the samples named as VHH-0 were set as negative controls, representing VHHs without modification but which had experienced all the physical treatment steps just same as modified VHHs. To show the binding kinetics and affinity change intuitively, on-rate (*k_a_*), off-rate (*k_d_)* and affinity constant (*K_D_*) of modified and unmodified VHHs were obtained by fitting a series of curves (Table S1). 

**Figure 4:**
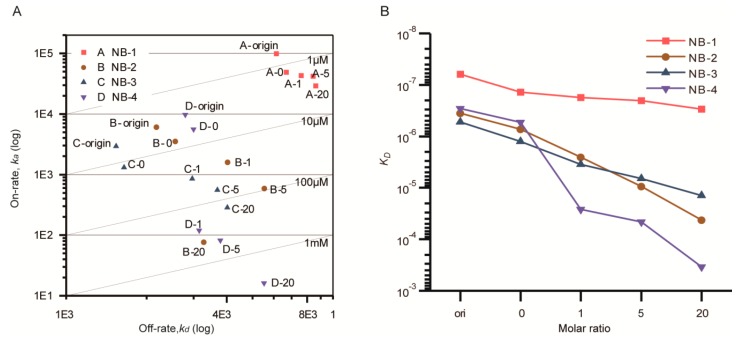
On-Off rate map (**A**) and affinity variation (**B**) of the modified VHH measured by Biacore T200. (**A**) Double logarithm coordinate was used; the diagonal line was equal affinity line (*K_D_*). (**B**) Single logarithm coordinate was used to show the affinity variation directly. Different VHHs were distinguished by colors. Five samples were measured for each VHH. The point “origin” represented VHHs without any treatment, the point “0” was rated as negative control, the points “1, 5, 20” represented VHHs modified with given molar ratio of NHS-Fluo. The *k_a_* of NB-4 modified by a 20 fold amount of NHS-Fluo was outside the limits that can be accurately measured by the Biacore T200 system.

After that, the *k_a_* and *k_d_* were visualized in the double logarithm coordinate (Figure 4A). The *K_D_* were displayed using line charts (Figure 4B). The combination and dissociation sensorgrams of the modified VHHs at the same concentration were presented in Figure S1. Compared with the corresponding samples without any treatment, the *K_D_* of NB-1-0, NB-2-0, NB-3-0 and NB-4-0 about increased to two-fold, and both on-rate and off-rate were influenced, indicating that extra processing steps including incubation and ultrafiltration slightly impacted the affinity of VHHs.

After being modified by increased molar ratios of NHS-Fluo, the affinities of four VHHs were decreased to different degrees. Briefly, the *K_D_* of NB-1 was limitedly affected, increasing from 1.38 × 10^−7^ M (negative control) to 2.97 × 10^−7^ M (modified by a 20 fold amount of NHS-Fluo). The *K_D_* of NB-2 increased 58-fold, from 7.26 × 10^−7^ M (negative control) to 4.23 × 10^−5^ M (modified by a 20 fold amount of NHS-Fluo). The *K_D_* of NB-3 increased 11-fold, from 1.26 × 10^−6^ M (negative control) to 1.41 × 10^−5^ M (modified by a 20 fold amount of NHS-Fluo). By analyzing the kinetic properties (Table S1), it could be found that, the increase of *K_D_* were mainly caused by the increase of on-rate. This showed that the additional NHS-Fluo linked to VHHs slowed the forming of antibody-antigen complex. On the contrary, NB-4 nearly lost all activity when modified by equal amounts of NHS-Fluo, and remarkably, the average FAR of modified products was only 0.22. This result suggests that fluorescein induced VHH denaturation even when no covalent binding occurred.

### 2.5. Structure Changes of the Modified VHHs

**Figure 5:**
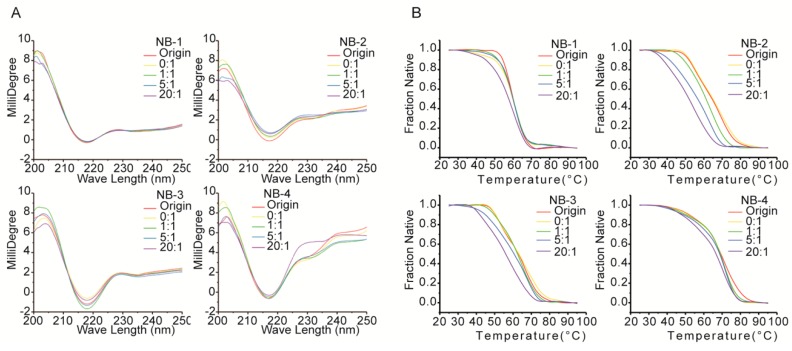
Spectra scanning (**A**) and thermal denaturation (**B**) of VHHs measured by CD. Five samples were taken for each VHH. The group “origin” represented original sample that without any treatment, the others groups represented the samples modified with corresponding molar ratio of NHS-Fluo. The spectrum signals at 216 nm were exported for thermal denaturation analysis.

To identify the secondary structural changes resulted from amino modifications, the original and modified proteins were evaluated by far-UV CD spectra (Figure 5A). On the one hand, the far-UV CD spectra of all VHHs had positive peaks in the range of 200–205 nm and negative peaks in the range of 210–225 nm, and the latter is a characteristic of β-sheet structure predominantly found in immunoglobulins. On the other hand, the differences between the far-UV CD spectra of four VHHs were shown clearly, indicating the difference among the secondary structures of each VHH or the original and modified VHHs. For the spectra of NB-1, there is no significant difference between the five groups, showing no substantial changes in the secondary structure among original or modified Nb-1. NHS-Fluo had no significant effect on the structural properties of NB-1. When modified by increased molar ratios of NHS-Fluo, the spectra of NB-2 were accompanied by a faint red shift (2 nm) around 216 nm, and a simultaneous flattening of the spectra from 230 nm to 240 nm. These are evidences on slight conformational changes of NB-2, affected by NHS-Fluo. The spectra of modified NB-3 did not change obviously and has no significant shift or peak, indicating NB-3 is more stable than NB-2. The spectra of modified NB-4 were more dissimilar than those of NB-1, NB-2 and NB-3. On one hand, like NB-2, the peak at 216 nm shifted slightly and the spectra flattened out from 230 nm to 240 nm. On the other hand, the positive peaks around 200 nm were dissimilar. It was clear from the drastic changes in the spectra that the amino modification affected the structure of NB-4 significantly. It is important to note that these results are consistent with the decrease of stability and bioactivity resulting from amino modification (Figure 3 and Figure 4).

The effect of amino modification on the thermostability of four VHHs was evaluated by thermal denaturation analysis. The data in Figure 5B illustrate the changes in CD millidegree of different VHHs with increasing temperature from 25 to 90 °C at 216 nm, where the characteristics of β-sheet structure predominate. Results showed only a single transition for samples of NB-1, NB-2 and NB-3 between 25 and 90 °C, but two transitions for samples of NB-4. The midpoints of the denaturation profiles were estimated to characterize the thermostability changes of four VHHs (Table 3). As modified by increased molar ratios of NHS-Fluo, NB-2 showed the biggest drop in denaturation temperature, followed by NB-3, and NB-1 that maintained good thermostability after modification. As observed from these results, the amino group modified products of NB-1 exhibited better resistant secondary structure against changes in temperature than NB-2 and NB-3. Denatured in a dissimilar pattern, the thermostability of NB-4 could not compare with NB-1, NB-2 and NB-3 directly.

### 2.6. Fluorescence Imaging by Laser Scanning Confocal Microscope

**Figure 6:**
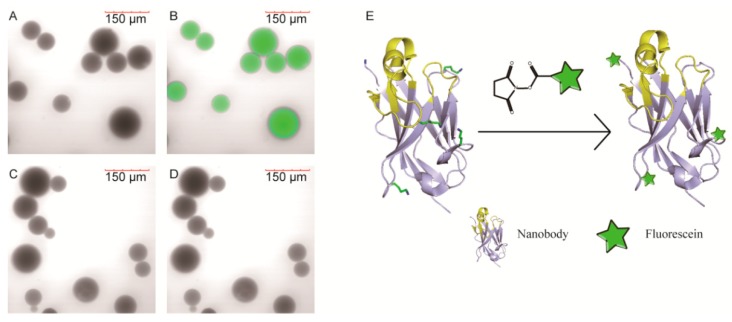
Labeled NB-1 stained beads visualized by laser scanning confocal microscope. (**A**,**B**) were β2m coated beads, (**C**,**D**) were BSA coated beads, (**A**,**C**) were bright field images, and (**B**,**D**) were merged images, (**E**) was the schematic diagram of VHHs modified by NHS-Fluo.

Fluorescence imaging was performed to verify the application potential of fluorescein labeled VHH. The merged image of β2m coated beads presented bright fluorescence (Figure 6B), in contrast, there was no fluorescence signal acquired from the merged images of BSA coated beads (Figure 6D). 

In biotechnology applications like fluorescence immunostaining and immunodetection, labeled antibodies are required to diffuse rapidly to tissue, and bind to antigens robustly and specifically. This system pattern can support highly that the labeled NB-1 could meet those requirements, as it diffuses rapidly to the porous structure, recognizes and binds to the β2m specifically.

## 3. Materials and Methods

### 3.1. Chemical Reagents

Antigen β2M was expressed and purified as previously described [27]. Proteinase trypsin and Glu-C, dithiothreitol (DTT) and iodoacetamide (IAM) were purchased from Thermo Scientific (MA, USA). Isopropyl β-d-thiogalactoside (IPTG) and 5-carboxyfluorescein N-succinimidyl ester (NHS-Fluo) were purchased from Beijing Solarbio Science & Technology Co., Ltd. (Beijing, China).

### 3.2. Production and Purification of VHHs

To construct the expression vectors, the anti-β2M VHH coding sequences were cloned in modified PET-21a vectors, and the flexible (G4S)4 linker was inserted between the VHH domain and 6xHis-tag, resulting in VHH-(G4S)_4_-His_6_. *E. coli* SHuffle^®^ T7 cells (NEB) were transformed with the plasmids for the standard protein expression. The transformed cells were cultured in 200 mL of TB medium at 37 °C for 5 h, and then induced with 5 mM IPTG at 18 °C for 20 h. The cells were harvested by centrifugation at 4000× *g* for 20 min at 4 °C, and then resuspended in 20 mM PBS buffer (pH 7.4) containing 500 mM NaCl. Homogenization was performed by an APV2000 high pressure homogenizer (APV, NC, USA). Supernatant was collected by centrifugation at 4000× *g* for 20 min at 4 °C. Followed closely, the interested VHHs were purified by affinity chromatography using the HisTrap HP column (GE Healthcare, MA, USA). In detail, the column was flowed by supernatant, and then washed with 20 mM PBS (pH 7.4) containing 500 mM NaCl and 20 mM imidazole. VHHs were eluted with 20 mM PBS (pH 7.4) containing 500 mM NaCl and 500 mM imidazole. Ultrafiltration of the eluate was carried out by Ultra Centrifugal Filters (Merck, Darmstadt, Germany) to exchange the protein storage buffer to 20 mM PBS (pH 7.4) with 150 mM NaCl. After analyzed by SDS-PAGE under reducing conditions the purified VHHs were stored at −80 °C for subsequent application.

### 3.3. Structure Homology-Modelling of VHHs

In order to show the amino group distribution in stereoscopic structure, homology-modelling was performed by SWISS-MODEL [28,29,30,31]. The structure with highest global model quantity estimation (GMQE) was employed as template for homology-modelling. After that, figures were prepared using Yasara [32]. The amino acid was numbered in Kabat scheme.

### 3.4. NHS-Fluo Modification of VHHs

Four VHHs were diluted by PBS (20 mM with 150 mM NaCl, pH 7.4) at the final concentration of 0.2 mM. Different molar ratio (0, 1, 5 or 20) of NHS-Fluo (5-carboxyfluorescein N-succinimidyl ester) to VHHs was added and the mixtures were incubated for 2 h at 4 °C. It should be noted that 20 folds of NHS-Fluo to VHHs was supersaturated in solution. The residual NHS-Fluo was removed by ultrafiltration. The final products were analyzed by native polyacrylamide gel electrophoresis (Native-PAGE) and fluorescence signal was visualized on ChemiDoc XRS+ system (Bio-Rad, CA, USA). After, the gel was stained with Coomassie brilliant blue and visualized.

### 3.5. Intact Protein Analysis by Mass Spectrometry

Intact protein analysis was performed to detect the Fluo-antibody ratio (FAR) and isomers composition of the modified VHHs. LTQ Orbitrap liquid chromatography-tandem mass spectrometry (LC-MS, Thermo Scientific) was used for mass spectrometry analysis. Briefly, 10 μL of intact protein (0.5 mg/mL) was injected and separated by a Hypersil GOLD™ C18 liquid chromatography column (Thermo). The mobile phase was acetonitrile-water, using acetonitrile linear gradient elution from 20% to 80% in 30 min with the column temperature 30 °C, followed by electron spray ionization mass spectrometry (ESI-MS). Xcalibur and BioPharma Finder Mass Informatics Platform (Thermo Scientific) were used for spectrum deconvolutions of protein characterization. Default SW Xtract (sliding windows and isotopically resolved) processing method was used as intact protein analysis definition, with *m/z* range of 200~2000 and merge tolerance 30 ppm.

### 3.6. Bioactivity Measured by Surface Plasmon Resonance

Kinetic and affinity measurements were performed on a Biacore T200 (GE Healthcare, MA, USA). The antigen β2M was immobilized on a CM5 chip to 150 response units (RU). The blank channel used as a subtraction was blocked by ethanolamine. Before and after modifications, samples were analyzed in general approach. Briefly, flow rate of 2-fold serial diluted VHH was 30 μL/min in HBS•EP+ and the chip was regenerated using 10 mM glycine-HCl (pH 1.5). All kinetic experiments were performed at 25 °C. Binding curves were fitted using the default ‘1:1 (antigen:analyte) with drift and RI2’ binding model in Biacore T200 evaluation software.

### 3.7. Far-UV Latter Spectra and Stability Analysis of VHHs

MOS-500 (Bio-Logic, Seyssinet-Pariset, France) was used for CD spectra scanning. Samples were diluted by 20 mM phosphate buffer (pH 8.0) with the final concentration of 0.05 mg/mL. Far-UV CD spectra were obtained in a 10 mm path-length cell at room temperature. The wavelength range of scanning was 190~250 nm, with a step size of 1 nm and three scans averaged for each CD spectrum. The curve was smoothed in BioKine, using Savitzky-Golay mathematics, 15 window pointsf, polynomial order 3. Secondary structures of VHHs were analyzed in Dicroprot, fitted by Contin (Linear Combination).

MOS-500 and TCU-250 (Bio-Logic) with a 10 mm path-length cell were used in combination for denature protein analysis and calculation of denaturation temperature. The temperature range was between 25 °C to 95 °C with a step size of 1 °C and temperature error of 0.5 °C. Before each measurement, wait for 1 min to equalize the temperature of samples. The wavelength range of the measurement was 190~250 nm, with a step size of 1 nm and three scans averaged for CD spectra of each temperature. After that, the curve of ellipticity change with temperatures rise in 216 nm was exported, and smoothed in Origin 2017, using Savitzky-Golay arithmetic, with 15 points of window, polynomial order 2. The fraction of native protein was calculated by formula (F1):

(F1) $$F_{N}=\frac{y_{t}-y_{u}}{y_{N}-y_{u}}$$

In the formula, *F_N_* represents the fraction of native protein, *y_t_* represents the optical rotation value in temperature t, *y_u_* represents the optical rotation value of completely denatured protein, and *y_N_* represents the optical rotation value of native protein.

### 3.8. Fluorescence Imaging by Laser Scanning Confocal Microscope

The antigen β2M or negative control - BSA were immobilized onto CNBr-activated Sepharose 4 Fast Flow media (GE Healthcare) following the recommended procedure. Briefly, the protein was dissolved into a coupling buffer, composed of 0.1 M NaHCO_3_ (pH 8.3) and 1 M NaCl with the concentration was 5 mg/mL. 0.1 g gel was resuspended by 0.5 mL of protein and stirred 3 h at room temperature. After that, excess protein was washed and non-reacted groups on the gel were blocked The protein coated beads were co-incubated with equal volume of labeled NB-1 (modified by a 20 fold amount of NHS-Fluo, 10 μg/mL in PBS) and stirred 5 min at room temperature, and then, the beads were adequately washed with 5 mL PBS for five times. After that, the beads were visualized on a FV1000 microscope (Olympus, Tokyo, Japan).

## 4. Conclusions

In this study, we characterized the influence of four selected VHHs modified by fluorescein on their amino groups. Several parameters were used to characterize the modified VHHs which were important for VHHs’ applications, including the protein aggregation, isomer composition, decreased bioactivity, structure changes and thermostability. The results fully demonstrated the individual variations of VHHs after amino modification, however, our analysis did not explain precisely which characteristics made VHHs robust during amino modification due to the complexity of VHHs. In particular, we found at last one VHH that retained its structural stability, antigen binding ability and thermostability after modification by NHS-Fluo, and a high titer of labels with good homogeneity was achieved. The labeled VHHs presented good performance in fluorescence imaging. Accordingly, amino modification could be a good option for VHH functionalization.

## Figures and Tables

**Table 1:** The statistic of lysine residues’ location in the selected four VHHs.

VHH	H43 ^[a]^	H64	H68	H75	H83	H100	H105	Model ID ^[d]^	GMQE
NB-1	K ^[b]^	K	X ^[c]^	K	X	K	X	4KDT	0.99
NB-2	K	K	X	X	K	X	X	5IML	0.92
NB-3	K	K	K	K	K	X	K	5J56	0.93
NB-4	K	X	X	X	X	X	X	5IMK	0.85

^[a]^ The amino acid was numbered in Kabat scheme (Hn) instead of the real sequence number. ^[b]^ “K” present lysine residue. ^[c]^ “X” present the rest of residues. ^[d]^ The PDB ID of the template selected for homology-modeling and its GMQE was listed.

**Table 2:** Average FAR and standard deviation of the modified VHHs.

Molar Ratio ^[a]^	NB-1		NB-2		NB-3		NB-4	
	FAR	SD	FAR	SD	FAR	SD	FAR	SD
1:1	0.25	0.52	0.44	0.84	1.02	1.30	0.22	0.43
5:1	1.21	1.03	1.29	1.25	2.68	1.71	0.71	0.85
20:1	1.83	1.25	2.79	1.35	4.91	1.90	1.29	0.85

^[a]^ The ratio of NHS-Fluo to VHH, in molar concentration.

**Table 3:** Midpoints of the denature profiles of the modified VHHs.

Molar Ratio	NB-1(°C)	NB-2(°C)	NB-3(°C)	NB-4(°C)
Original	57.5	58.5	63.5	69.5
0:1	57.0	58.0	63.0	69.0
1:1	56.5	56.0	62.5	69.0
5:1	56.0	50.0	60.5	68.5
20:1	53.0	45.5	56.0	66.5

## Supplementary Materials

The following are available online at https://www.mdpi.com/1420-3049/24/14/2600/s1, Figure S1. The combination and dissociation sensor grams of the different molar ratio modified VHHs in the same concentration. Table S1. The kinetic properties of modified VHHs.
