# Supplementary material for: Generation and Application of Fluorescent Anti-Human β2-Microglobulin VHHs via Amino Modification
Source: Molecules. 2019 Jul 17;24(14):2600. doi: 10.3390/molecules24142600 (PMC6680903; doi:10.3390/molecules24142600)
Supplement: Supplementary file 1 [file molecules-24-02600-s001.pdf]

Supporting information

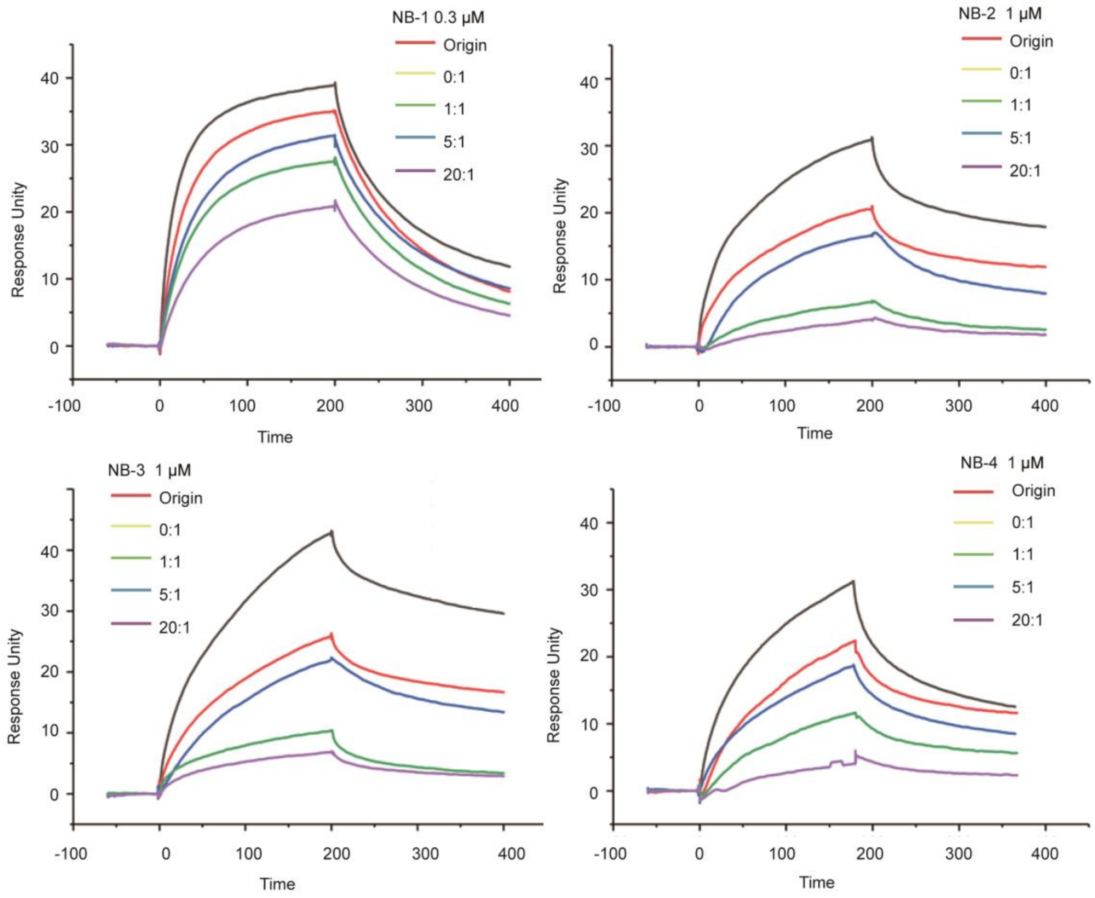

Fig. S1. The combination and dissociation sensor grams of the different molar ratio modified VHHs in the same concentration. The group “origin” represented the original sample that without any treatment, the others groups represented the samples modified with corresponding molar ratio of NHS-Fluo.

Tab. S1. The kinetic properties of modified VHHs.

Sample[a]	NB-1-Origin	NB-1-0	NB-1-1	NB-1-5	NB-1-20
k_a (1/Ms)	9.93E+04	4.85E+04	4.30E+04	4.18E+04	2.90E+04
k_d (1/s)	6.14E-03	6.69E-03	7.61E-03	8.43E-03	8.63E-03
K_D (M)	6.18E-08	1.38E-07	1.77E-07	2.02E-07	2.97E-07

Sample	NB-2-Origin	NB-2-0	NB-2-1	NB-2-5	NB-2-20
k_a (1/Ms)	6.12E+03	3.54E+03	1.60E+03	5.91E+02	7.74E+01
k_d (1/s)	2.18E-03	2.57E-03	4.03E-03	5.54E-03	3.28E-03
K_D (M)	3.56E-07	7.26E-07	2.53E-06	9.37E-06	4.23E-05

Sample	NB-3-Origin	NB-3-0	NB-3-1	NB-3-5	NB-3-20
k_a (1/Ms)	2.94E+03	1.31E+03	8.49E+02	5.58E+02	2.86E+02
k_d (1/s)	1.54E-03	1.65E-03	2.97E-03	3.69E-03	4.02E-03
K_D (M)	5.25E-07	1.26E-06	3.49E-06	6.62E-06	1.41E-05

Sample	NB-4-Origin	NB-4-0	NB-4-1	NB-4-5	NB-4-20
k_a (1/Ms)	9.70E+03	5.60E+03	1.20E+02	8.21E+01	1.62E+01
k_d (1/s)	2.80E-03	3.00E-03	3.15E-03	3.79E-03	5.52E-03
K_D (M)	2.89E-07	5.36E-07	2.62E-05	4.62E-05	3.42E-04

[a] The samples “NB-1-Origin” represented the original NB-1 that without any treatment, the samples “NB-1-0” represented the negative control, and the samples “NB-1-1, NB-1-5, NB-1-20” represented the NB-1 modified with 1, 5, 20 times of NHS-Fluo. The other samples were named in the same way.
